# Evaluation of score-based tertiary triage policies during the COVID-19 pandemic: simulation study with real-world intensive care data

**DOI:** 10.1007/s00063-024-01162-8

**Published:** 2024-08-02

**Authors:** Christina C. Bartenschlager, Jens O. Brunner, Michael Kubiciel, Axel R. Heller

**Affiliations:** 1 Applied Data Science in Healthcare, Nürnberg School of Health, Ohm University of Applied Sciences Nuremberg, 90489 Nürnberg, Germany; 2 Anaesthesiology and Operative Intensive Care Medicine, Faculty of Medicine, University of Augsburg, University Hospital of Augsburg, Stenglinstraße 2, 86156 Augsburg, Germany; 3 Decision Science in Healthcare, Department of Technology, Management, and Economics, Technical University of Denmark, Akademivej, Kongens Lyngby, 2800 Denmark; 4 Chair of German, European and International Criminal, Medical and Economic Law, University of Augsburg, Universitätsstraße 24, 86159 Augsburg, Germany

**Keywords:** Pandemics, Triage, Intensive care unit, Simulation, Real-world data, Pandemie, Triage, Intensivstation, Simulation, Real-World-Daten

## Abstract

**Objective:**

The explicit prohibition of discontinuing intensive care unit (ICU) treatment that has already begun by the newly established German Triage Act in favor of new patients with better prognoses (tertiary triage) under crisis conditions may prevent saving as many patients as possible and therefore may violate the international well-accepted premise of undertaking the “best for the most” patients. During the COVID-19 pandemic, authorities set up lockdown measures and infection-prevention strategies to avoid an overburdened health-care system. In cases of situational overload of ICU resources, when transporting options are exhausted, the question of a tertiary triage of patients arises.

**Methods:**

We provide data-driven analyses of score- and non-score-based tertiary triage policies using simulation and real-world electronic health record data in a COVID-19 setting. Ten different triage policies, for example, based on the Simplified Acute Physiology Score (SAPS II), are compared based on the resulting mortality in the ICU and inferential statistics.

**Results:**

Our study shows that score-based tertiary triage policies outperform non-score-based tertiary triage policies including compliance with the German Triage Act. Based on our simulation model, a SAPS II score-based tertiary triage policy reduces mortality in the ICU by up to 18 percentage points. The longer the queue of critical care patients waiting for ICU treatment and the larger the maximum number of patients subject to tertiary triage, the greater the effect on the reduction of mortality in the ICU.

**Conclusion:**

A SAPS II score-based tertiary triage policy was superior in our simulation model. Random allocation or “first come, first served” policies yield the lowest survival rates, as will adherence to the new German Triage Act. An interdisciplinary discussion including an ethical and legal perspective is important for the social interpretation of our data-driven results.

**Supplementary Information:**

The online version of this article (10.1007/s00063-024-01162-8) contains supplementary material, which is available to authorized users.

## Abstract (de)

**Ziel:**

Das Verbot, unter Krisenbedingungen eine bereits begonnene Behandlung auf der Intensivstation (ICU) zugunsten neuer Patienten mit besserer Prognose zu beenden (Tertiärtriage), durch die neue gesetzliche Regelung zur Triage könnte verhindern, dass so viele Patienten wie möglich gerettet werden, und verletzt daher möglicherweise die international anerkannte Prämisse, das „Beste für die Meisten“ Patienten zu tun. Während der COVID-19-Pandemie verordneten die Behörden Lockdown-Maßnahmen und Infektionspräventionsstrategien, um eine Überlastung des Gesundheitswesens zu verhindern. In Fällen einer situativen Überlastung der Intensivkapazitäten, wenn die Abverlegungsmöglichkeiten erschöpft sind, stellt sich die Frage nach einer tertiären Triage von Patienten.

**Methoden:**

Es werden datenwissenschaftliche Analysen von scorebasierten und nicht scorebasierten tertiären Triagestrategien unter Verwendung von Simulation und realen elektronischen Gesundheitsdaten in einer COVID-19-Situation dargestellt. Dabei werden 10 verschiedene Triagestrategien, z. B. auf der Grundlage des Simplified Acute Physiology Score (SAPS II), anhand der resultierenden Mortalität auf der ICU und inferenzstatistschen Methoden verglichen.

**Ergebnisse:**

Die vorliegende Studie zeigt, dass scorebasierte tertiäre Triagestrategien zu einer geringeren Mortalität führen als ein nicht scorebasierter Ansatz wie er in der deutschen Triageregelung festgelegt ist. Auf der Grundlage des hier verwendeten Simulationsmodells verminderte eine auf dem SAPS-II-Score basierende tertiäre Triagestrategie die Mortalität auf der ICU um bis zu 18 Prozentpunkte. Je länger die Schlange intensivpflichtiger Patienten, die auf einen ICU-Platz warten, und je größer die Maximalzahl von Patienten, die der tertiären Triage unterliegen, desto größer ist die Wirkung auf die Reduktion der Mortalität auf der ICU.

**Schlussfolgerung:**

Eine auf dem SAPS-II-Score basierende tertiäre Triagestrategie war in dem hier verwendeten Simulationsmodell überlegen. Die zufällige Verteilung oder Strategien wie „first come, first served“, führten zu den höchsten Mortalitätsraten, ebenso wie auch die Befolgung der neuen Triageregelung. Eine interdisziplinäre Diskussion einschließlich einer ethischen und rechtlichen Perspektive ist wichtig für die gesellschaftliche Interpretation der vorliegenden datenwissenchaftlichen Ergebnisse.

## Highlights.

- We analyze the efficiency of different forms of score- and non-score-based tertiary triage policies in a COVID-19 setting based on real-world intensive care data.
- We find that score-based tertiary triage policies outperform non-score-based tertiary triage policies. Random allocation or a “first come, first served” policy leads to the lowest survival rates, as will adherence to the new German Triage Act.
- The results of our study can be relevant for authorities, researchers, decision-makers in hospitals, the current political decision-making process, and evidence-based legislation.

## Introduction

With the coming into force of Section 5c of the German Infection Protection Act (IfSG), the so-called Triage Act, on 14 December 2022, a heated discussion has come to a provisional conclusion, with the result of which doctors, but also lawyers and ethicists, are equally dissatisfied with [1, 2]. Triage describes a selection process concerning the allocation of scarce medical resources to patients. For triage of intensive care patients, a distinction is made between ex ante and tertiary triage. In ex ante triage, a selection of patients before admission to the intensive care unit (ICU) is necessary because more patients are waiting for intensive care treatment than can be admitted to the ICU. In the literature, *tertiary triage*, as described by Christian [3] and sometimes also denoted as ex post triage [4] or reverse triage [5], is not defined consistently. Generally, the term indicates that patients already treated in the ICU are included in the consideration for triage. The explicit prohibition on discontinuing treatment that has already begun in favor of new patients with better prognoses (tertiary triage) prevents allocation decisions, with the aim of saving as many patients as possible under crisis conditions [6, 7]. Besides others, both the German Medical Association [8] and the Association of the Scientific Medical Societies in Germany [9] have clearly committed themselves to the international premise of undertaking the “best for the most” patients [3, 10] in their statements on Section 5c IfSG in order to save as many lives as possible. This is one of the reasons why several constitutional complaints have been lodged against Section 5c IfSG.

The relevance of ex post triage under criminal law is disputed among lawyers. While traditional lawyers call for a random principle or a “first come, first served” approach and state that ex post triage is punishable, renowned representatives of the field cast doubt on this [11, 12]. In order to safeguard the legitimate interest of physicians in not being targeted by law enforcement authorities in the event of a need for triage through no fault of their own, empirical proof of the usefulness of improved survival rates through the use of particular ex post triage policies based on a broad data basis is still lacking.

During the COVID-19 pandemic, authorities set up strict lockdown measures and infection-prevention strategies to avoid the scenario of an overburdened health-care system. However, several hospitals were on the verge of running out of intensive care resources especially throughout the fourth pandemic wave in 2021 [13] and in particular when patient transport capacities were exhausted. In cases of situational overload of intensive care resources, the question of patient triage arises.

Triage policies aim to use medical resources as efficiently as possible. In the ethical and legal debate, it is disputed which parameters may be taken into account in a decision: While some argue for the consideration of the patient’s age, others focus primarily on medical criteria such as medical scores, e.g., the Simplified Acute Physiology Score (SAPS II); still others consider random-based methods preferable because they promise to give all patients equal chances [14]. Although triage is to be understood as ultima ratio, tertiary triage in particular remains ethically controversial and does define an important research stream.

Compared to primary (ex ante) triage, tertiary triage is a rather young research area [5], which is dealt with primarily from a theoretical, ethical, and legal perspective (see, e.g., [15, 16]). Related data-driven research focuses on machine learning approaches (see, e.g., [17, 18]), early discharge (see, e.g., [19]), the comparison of different score-based reverse triage approaches (see, e.g., [20]), the comparison of risk scores (see, e.g., [21]), Markov decision processes (see, e.g., [22]), the evaluation of ICU management policies by simulation (see, e.g., [23, 24]), the evaluation of age-based tertiary triage by simulation (see, e.g., [25]), or the evaluation of ICU triage for disabled people [26]. Thereby, considerations include COVID-19 (see, e.g., [17, 18, 23, 25, 26]) and non-COVID-19 settings (see, e.g., [19–22, 24]) and vary in the definition of triage. Most contributions concentrate on a definition of tertiary triage with an option for (early) discharge.

In our pre-study [13], we provide a data-driven evaluation of score- and non-score-based tertiary triage policies using simulation, real-world data, and a COVID-19 setting without an option for (early) discharge. The study was of an ad hoc nature with a 1-day database, one tertiary triage point in time only, and hand-crafted data preparation. We tackle these drawbacks of our pre-study and present the results of broad simulation analyses based on a database including all intensive care patients of the University Hospital of Augsburg, Germany, during the fourth pandemic wave, automated data preparation, and three tertiary triage points in time to support authorities, researchers, the formation of opinion in society as a whole and the German Federal Constitutional Court, as well as decision-makers in hospitals.

## Methods

### Data preparation

A data export of the hospital information system of the University Hospital of Augsburg including all intensive care patients starting from 1 September 2021 to 31 December 2021 (fourth pandemic wave in Germany) was the basis for our study. Data were provided in automatically pseudonymized form by the trust center of the data integration center of Augsburg University Hospital. The pseudonymized raw data included the age of the patient, the SAPS II [27] and Therapeutic Intervention Scoring System (TISS; [28]) scores during the hospital stay, the main diagnosis, secondary diagnoses, start dates and end dates of the ICU stay and the hospital stay, and information on whether the patient died during the hospital stay. As the data structure did not fit the data structure needed for the simulation study, data preparation was necessary. Data manipulation focused on the selection of the first SAPS II and TISS scores and the identification of the COVID-19 diagnosis based on automated text analyses and International Statistical Classification of Diseases and Related Health Problems (ICD)-10 codes . In addition, we automatically calculated the length of stay (LOS) and the number of secondary diagnoses based on the raw data. In the case of more than one ICU stay during the hospital stay, we included the first ICU stay only. In a second step, the data of patients with implausible entries according to the number of main diagnoses, missing values, or negative LOS were deleted.

For the remaining patients, we calculated the triage score according to the recommendations of the respective practice guidelines by the German Interdisciplinary Association for Intensive Care and Emergency Medicine (DIVI) [6, 29]. We calculated an adjusted version of the DIVI score: The original DIVI score considers the Sequential Organ Failure Assessment (SOFA) score [30], a prognosis regarding limiting factors, and age as a secondary criterion. The points for the different criteria per patient are specified by the triage team of the hospital and summed up. Since the SOFA score is not commonly stored in the hospital information system of the University Hospital of Augsburg, but the SAPS II score is, we adjusted the respective point scale of the SOFA score for the SAPS II score, based on a comparison of the maximum values (SOFA: 24, SAPS II: 163) of both scores [31] were able to show in COVID-19 patients that the SAPS II score had higher precision than the SOFA score in terms of predicting mortality. The prognosis regarding the limiting factors criterion was substituted by the number of secondary diagnoses and a detailed discussion with physicians experienced in intensive care (see Supplementary Table 1). For the evaluation of our simulation results, we calculated the SAPS II-predicted mortality rate per patient as proposed by Le Gall et al. ([27]; see Supplementary Description 1).

### Tertiary triage policies

**Fig. 1:**
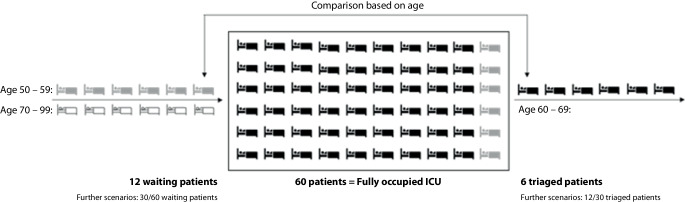
Example of age-based tertiary triage for a scenario with 12 patients waiting in the queue and a fully occupied ICU (60 patients). Based on a comparison of the ages of the patients waiting in the queue and those in the ICU, six patients waiting in the queue (*highlighted in gray*) are admitted to the ICU and six patients (*highlighted in black*) are discharged from the ICU. Six patients in the queue (*highlighted in white*) are not admitted to the ICU

In tertiary triage, we focus on a fully occupied ICU. Consequently, on the one hand, an ICU patient might be discharged, because another patient is waiting in the queue. On the other hand, a patient waiting in the queue might not be admitted to the ICU. The decision on discharge of existing intensive care patients and admission of critical care patients waiting in the queue is based on tertiary triage policies (see Fig. 1). In our study, we evaluated ten different criterion- and non-criterion-based tertiary triage policies. Criterion-based policies encompassed objective criteria such as age or predefined scores. Score-based policies were criterion-based policies including medical scores. Non-criterion-based policies involved random selection, for example. Table 1 summarizes the ten tertiary triage policies.

**Table 1:** Description of the tertiary triage policies *p*. In the case of policy 2 (first come, first served, FCFS), existing ICU patients are triaged based on a “reverse” FCFS concept

Triage policy number *p*	Rationale of the triage policy	Non-criterion-based policy	Criterion-based policy	Score-based policy
0		✓		
1	Random triage of patients	✓		
2	First come, first served (FCFS)-based triage of patients	✓		
3	Age-based triage of patients		✓	
4	SAPS II-based triage of patients		✓	✓
5	TISS-based triage of patients		✓	✓
6	Number of secondary diagnoses-based triage of patients		✓	
7	ICU length-of-stay (LOS)-based triage of patients		✓	
8	Adjusted DIVI score-based triage of patients without age		✓	✓
9	Adjusted DIVI score-based triage of patients with age		✓	✓

*DIVI* German Interdisciplinary Association for Intensive Care and Emergency Medicine, *SAPS* Simplified Acute Physiology Score, *TISS* Therapeutic Intervention Scoring System

An effective triage policy, for example, based on age as illustrated in Fig. 1, leads to lower overall mortality on the ICU than is caused by no ex post triage. This is due to the fact that more patients are treated in the ICU that actually benefit from intensive care treatment. An ineffective triage policy, for example, based on random selection, leads to equal or higher overall mortality in the ICU than is caused by no ex post triage.

### Simulation study and evaluation

For the simulation study in the software R, we defined an ICU capacity of 60 patients, because the University Hospital of Augsburg treated a maximum of 60 ICU patients (rounded) at the same time during the fourth pandemic wave. The number of consecutive tertiary triage points in time, e.g., days, was set to 3; that is, in timepoint $t=1$ (e.g., day 1), $t=2$ (e.g., day 2), and $t=3$ (e.g., day 3) tertiary triage was applied. We varied the number of critical care patients waiting in the queue by 12, 30, and 60. Due to the significant effort associated with a transfer of one patient and an immediate readmission of another patient, we assumed a maximum number of existing ICU patients subject to tertiary triage. We varied the maximum number of existing intensive care patients subject to tertiary triage by 6, 12, and 30. The combination of number of critical care patients waiting in the queue and maximum number of existing intensive care patients subject to tertiary triage defined the six different scenarios in our simulation study (see Supplementary Tables 2 and 3). For example, scenario 1 was defined by 12 critical care patients waiting in the queue and a maximum number of 6 existing intensive care patients subject to tertiary triage (see Fig. 1).

For every scenario, we simulated 1000 occupancies of the ICU and three consecutive queues by randomly sampling patients with replacement. For every existing intensive care patient, we randomly generated the currently elapsed time in the ICU in $t=1$ from a discrete uniform distribution. The existing intensive care patients eligible for tertiary triage and the patients in the queue were compared based on the applied tertiary triage policy, e.g., based on age. For every tertiary triage policy and timepoint, the average mortality in the ICU and the average SAPS II-predicted mortality in the ICU for the 1000 simulation runs were calculated. The mortalities were inferentially statistically compared by analyses of variance (ANOVAs) and post hoc tests with a 5% significance level. We applied Tukey’s honestly significant difference (HSD) post hoc test [32]. A policy was assumed to be superior if the corresponding mortality was smaller than the mortality calculated for another tertiary triage policy. A detailed description of the simulation study is provided in Supplementary Description 2.

### Research ethics

The study was conducted in accordance with the Declaration of Helsinki Ethical Principles and Good Clinical Practice. The responsible independent ethical review board of the Ludwigs Maximilians University (LMU) Munich reviewed the protocol (Ref. No. 22-0194 KB) and provided the investigators with written documentation that the study is exempt from further review. Individual informed consent of patients or legal representatives was waived.

## Results

**Fig. 2:**
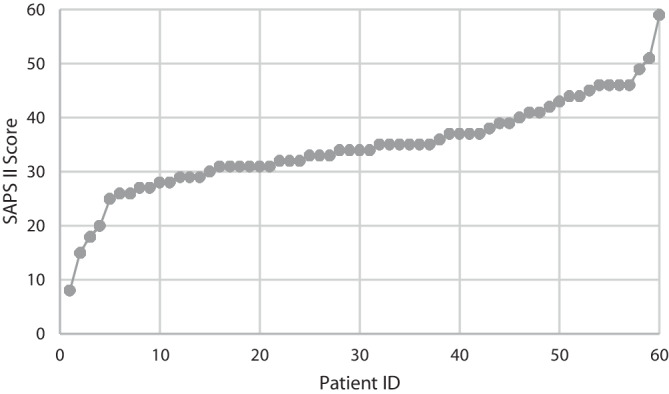
SAPS II distribution for all patients in the ICU on 1 December 2021. *SAPS* Simplified Acute Physiology Score

After data preparation and exclusion of four patients, we had a data set comprising 1083 patients and ten characteristics per patient (excl. DIVI scores and SAPS II-predicted mortality rate). The data set includes 247 COVID-19 and 836 non-COVID-19 patients with a mortality of 23.8%. In the given timeframe, approximately 64.0% of the ICU patients were male. Further demographic details can be found in Table 2. Non-surviving patients had the highest average SAPS II (44.0) and TISS scores (11.9) compared to COVID-19 patients, non-COVID-19 patients, and surviving patients. Figure 2 shows a representative SAPS II distribution over the 60 ICU patients on 1 December 2021 with identifiable outliers.

**Table 2:** Demographic characteristics of the cohort

	All patients
Number of patients	1083
Age (years)	64.6 ± 15.7
Comorbidities/secondary diagnoses (*n*)	12.2 ± 6.4
SARS-CoV‑2 (%)	22.8
SAPS	35.0 ± 12.7
TISS	9.4 ± 5.4
ICU-LOS (days)	4.0 ± 5.4
Hospital LOS (days)	17.8 ± 13.6

*LOS* length of stay, *SAPS* Simplified Acute Physiology Score, *TISS* Therapeutic Intervention Scoring System

The mean SAPS II-predicted mortality rate is lower than 0.5, i.e., 0.2, for all groups of patients, and 111 out of 1083 patients (10.3%) are predicted to die during their hospital stay based on the SAPS II-predicted mortality rate. While patients stay $17.8$ days in hospital, the average ICU-LOS is $4.0$ days. A histogram of the ICU-LOS is presented in Supplementary Fig. 1. In addition, the average number of secondary diagnoses for all patients is 12.2 and 14.5 for non-surviving patients (see Supplementary Table 4 for further details on the descriptive statistics of our data set).

Based on the simulation study, a policy of no tertiary triage (policy 0) leads to average mortality rates in the ICU of 23.6–24.1% and is comparable to the real-world mortality rate in the ICU for the full data set. Random (policy 1) and FCFS (policy 2) tertiary triage show average mortality rates of 22.6–24.1% depending on the scenario. The average mortality rates in the ICU of the criterion-based policies, i.e., policies 3 (age-based), 6 (number of secondary diagnoses-based), and 7 (ICU-LOS-based), vary from 22.6% (scenario 1 and $t=1$) to 9.4% (scenario 6 and $t=3$). For score-based policies, i.e., policies 4 (SAPS-based), 5 (TISS-based), 8 (adjusted DIVI-score-based without age), and 9 (adjusted DIVI-score-based with age), the values vary from 21.5% (scenario 1 and $t=1$) to 5.7% (scenario 6 and $t=3$). For clinical decision-making this means that criterion-based policies are superior to non-criterion-based policies such as FCFS-based policies.

**Fig. 3:**
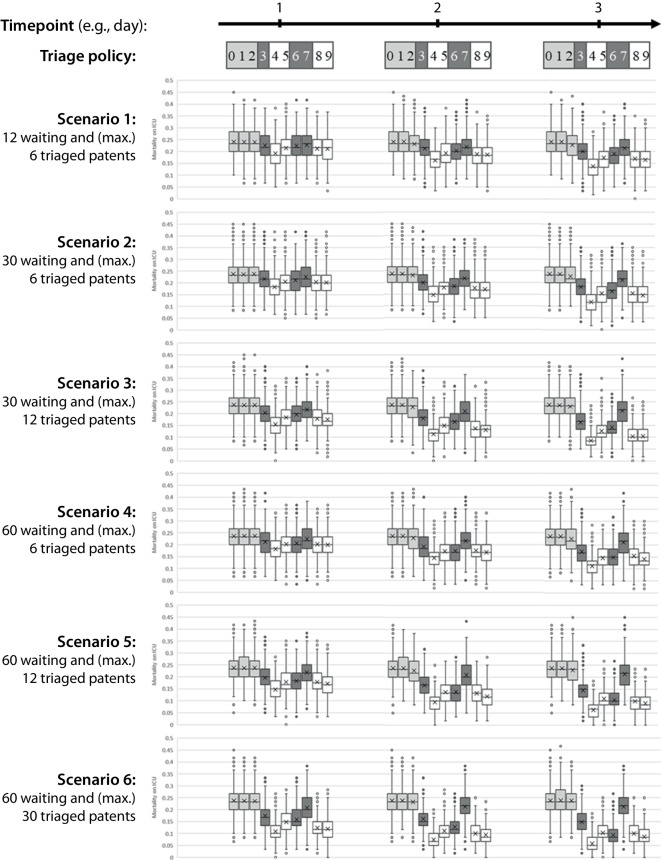
Boxplots for scenario 1 (*upmost*), 2, 3, 4, 5, and 6 (*lowermost*), timepoints $t=1$ (*left*), $t=2$ (*middle*), $t=3$ (*right*), and triage policies. *White* marks the score-based policies 4, 5, 8, and 9; *dark gray* marks the criterion-based policies 3, 6, and 7; and *gray* marks the non-score-non-criteria-based policies 0, 1, and 2. Descriptions of the policies are listed in Table 1

For SAPS II score-based tertiary triage (policy 4), we find the minima of average mortality rates int he ICU for all scenarios and points in time, while the global minimum is 5.7% (scenario 6 and $t=3$). Supplementary Table 5 shows the average mortality in the ICU for the different policies, points in time, and scenarios. Figure 3 provides the boxplots for all policies, points in time and scenarios and summarizes the distributions of the simulation outcomes. For clinical decision making this means, that SAPS II based policies are even superior to the TISS-based and DIVI-acore-based policies.

The application of the SAPS II-predicted mortality in the ICU leads to a systematic underestimation of the realized mortalities in the ICU, while the interpretation regarding the optimal policy and the performance of policies remains unchanged (see Supplementary Table 6).

The inferential statistical analysis of our simulation results (see Supplementary Table 7) shows that for all points in time and scenarios, there are significant differences between the policies, i.e., all *F-*test *p* values are 0. Based on the post hoc *p* values, we find that the non-criterion-based policies, policies 1 (random) and 2 (FCFS), do not differ significantly from policy 0 (no ex post triage) in most settings but they differ from the remaining criterion-based and score-based policies. Most of the criterion-based policies, i.e., policies 3 (age-based), 6 (number of secondary diagnoses-based), and 7 (ICU-LOS-based), differ significantly from each other. The SAPS II score-based tertiary triage, policy 4, differs significantly from all other policies.

## Discussion

Particularly in the current phase of collecting evidence for the debate at the German Federal Constitutional Court on the legitimacy of Section 5c IfSG, the existence of empirical evidence that criteria-driven triage policies significantly increase the survival rate under crisis conditions [10] will be decisive for the assessment. This essentially includes the comparison of triage policies or the current legally prescribed abandonment of these policies [1, 2]. Ultimately, reliable data must be available for the forthcoming discourse on ex post triage in society as a whole.

We evaluated different triage policies with extensive simulation studies on real data of ICU occupancy at Augsburg University Hospital from a 4-month peak phase of the COVID-19 pandemic (fourth pandemic wave). Ten different ex post triage policies, among others based on the DIVI recommendation published here [6] or their exclusion, were compared on the basis of the resulting lethality in different hospital stress scenarios.

Our study shows that criterion-based tertiary triage has a superior performance compared with non-criterion-based tertiary triage, while score-based policies lead to the lowest mortality rates in the ICU. Random allocation or a “first come, first served” policy leads to the lowest survival rates, as will adherence to the new German Triage Act. Our findings are supported by an analysis of the Danish National Intensive Care Patient Database with more than 230,000 records, which shows that the inclusion of longer-term patient history in a score-based prediction model significantly improves prognostic accuracy [33]. The inferential statistical analysis of our results supports the differences in the policies. Overall, a SAPS II score-based tertiary triage is superior and reduces mortality in the ICU, depending on the scenario—i.e., the length of the queue, the maximum number of patients subject to tertiary triage, and the time horizon—by up to 18 percentage points. The integration on age in the DIVI score leads to a further reduction in the mortality in the ICU of 1 percentage point compared with the DIVI score without age, but the differences are not significant in most settings.

The longer the queue and the larger the maximum number of patients subject to tertiary triage, the greater the effect on the reduction of mortality in the ICU. From a mathematical perspective, this finding is supported by the law of large numbers and the preference for patients with a higher chance of survival in the triage process. However, one should consider that the maximum number of patients subject to tertiary triage has practical limits, because the transfer of a patient requiring intensive care and the simultaneous readmission of a new critical care patient lead to a high logistical effort. Regarding the evaluation of the influence of the time horizon, our analyses show that the application of tertiary triage over time guarantees significant further reduction in the mortality in the ICU.

The use of the SAPS II-predicted mortality in the ICU as a performance metric indicates a systematic underestimation of the realized mortalities in the ICU based on the descriptive statistical analysis of the data set and our simulation model. This observation was confirmed by the authors of the SAPS II score when it was re-evaluated 12 years after the initial publication [34]. They showed in a group of 77,490 patients that mortality in newer patient cohorts is underestimated by the SAPS II-based formula. The reason for this is the fact that today patients have more comorbidities and a higher age. This is especially true in the cohort of COVID-19 patients requiring intensive care, many of whom were older, overweight, and had diabetes [35]. As the authors of the SAPS II score themselves acknowledge in more recent studies [34], our data underline the need for an updated version of the calculation of the mortality rate predicted by the SAPS II. However, this score is still suitable for ranking within a given cohort (see, e.g., [31, 36]). Thus, the comparisons in our study are not affected by this score-to-mortality conversion issue and are still valid when keeping interpretations within the comparison frame of the score itself. In this context, it has been criticized that a specific score may not be able to distinguish between individual patients with sufficient certainty during triage [37] and that the legitimacy of ex post triage is questionable if even a slightly better prognosis could lead to a reallocation of intensive care resources. In this respect, the standard for reallocation should be formulated particularly strictly [11]. However, a bird’s-eye view of all patients in a cohort (Fig. 2) makes it possible to identify people at the thinner ends of the scale with considerable differences in prognosis. This view makes it possible to draw attention, on the one hand, to patients whose good condition enables safe downgrading to a low-care ward or to recognize which patients need to be considered for ex post triage, on the other hand.

### Limitations

Our study is subject to some limitations, described in the following. First, we evaluated tertiary triage over time. We did so by the assumption of no discharge in our time horizon of *T* = 3 days and an expected ICU-LOS of 4 days. The orientation by the mean ICU-LOS enabled us to evaluate the effect of tertiary triage over time but it does not consider the skewness of the ICU-LOS distribution (see Supplementary Fig. 1). Second, in our simulation model, we did not consider the influence of active participation of the patient in the decision-making process, the influence of the actual clinical management of ICU patients, the influence of a change in therapy inside the ICU to palliative care, and the influence of living wills, which individually exclude hospitalization, admission to the ICU, intubation (DNI: do not intubate), or resuscitation (DNAR: do not attempt to resuscitate) etc. As this potentially affects all patients equally, we assume that the simulation result is not subject to a systematic bias. Fourth, we included the retrospective LOS in the ICU as a criterion for tertiary triage. One might wonder about this process, because the ICU-LOS might be unknown during the actual decision on the admission or withdrawal of a patient. However, throughout the COVID-19 pandemic, machine-learning approaches including a prediction of different patient characteristics were researched extensively. Thus, the ICU-LOS of a patient may be known at the time of a tertiary triage decision. In addition, an orientation on a short ICU-LOS supports the basic idea of catastrophic triage, i.e., “do the best for the most.” Fifth, in practice, the selection process should be based on a group of ethical and medical experts as various scientific societies suggest (see, e.g., [6]). The adaptation of the DIVI score for our data cannot fully replicate the extensive discussion of this triage team but reflects the unadorned figures that a triage team can use as the basis for its decision [38].

The findings of this research cannot be implemented in clinical practice under current legislation and instead fulfill the criminal offence of homicide. Therefore, also in crisis care [10] physicians should be particularly consistent in doing what is good clinical practice: Only carry out those medically indicated intensive care treatments that are (still) in the interest of the patients, i.e., that serve their well-being and are requested by them avoiding overtreatment with intensive care [7], in particular by regularly reviewing them. Before considering treatment restrictions due to a lack of ICU resources, all options for transporting patients to another suitable hospital must be fully utilized [1]. Decisions to limit or terminate life-sustaining intensive care measures must be documented exactly. At the same time, however, shortfalls in patient care, e.g., due to incorrectly underestimating the chances of survival in old, frail, chronically ill, or disabled patients, must be avoided [1].

## Conclusion

In this work, we evaluate non-criterion-based, criterion-based, and within the latter, score-based and non-score-based tertiary triage policies in a COVID-19 setting by a simulation study and real-world intensive care data of the fourth pandemic wave in Germany. We find that score-based tertiary triage policies are superior to non-score-based policies. Non-score-based policies perform better than non-criterion-based policies. Based on the simulation model, SAPS II score-based tertiary triage is superior. Random allocation or a “first come, first served” policy leads to the lowest survival rates, as will adherence to the new German Triage Act. The results of our study might be of particular importance for authorities, decision-makers in hospitals, and the political decision-making process in light of the pending constitutional lawsuit regarding the controversial German Triage Act. Under current German legislation the findings of our study may not directly be implemented in clinical practice [1].

### Take-home messages

We analyze the efficiency of different forms of score-based and non-score-based tertiary triage policies in a COVID-19 setting on the basis of a simulation study and real-world intensive care data. We find that score-based tertiary triage policies outperform non-score-based tertiary triage policies. Random allocation or a “first come, first served” policy leads to the lowest survival rates, as will adherence to the new German Triage Act. Under current German legislation, the findings of our study may not directly be implemented in clinical practice.

## Supplementary Information

The Supplementary Information includes detailed descriptions of the calculation of SAPS II-predictced mortality, of the proceeding of the simulation study and of the results of the simulation study.

## Data Availability

Due to data privacy, data cannot be made available via a repository. Please contact the corresponding author.

## Abbreviations

ANOVA: Analysis of variance
COVID-19: Coronavirus disease 2019
DIVI: German Interdisciplinary Association for Intensive Care and Emergency Medicine
FCFS: First come, first served
ICD-10: International Statistical Classification of Diseases and Related Health Problems 10
ICU: Intensive care unit
LMU: Ludwig Maximilians University
LOS: Length of stay
SAPS II: Simplified Acute Physiology Score II
SOFA: Sequential Organ Failure Assessment
TISS: Therapeutic Intervention Scoring System

## Declarations

For this article no studies with human participants or animals were performed by any of the authors. All studies mentioned were in accordance with the ethical standards indicated in each case. The responsible independent ethical review board of the LMU Munich has reviewed the protocol (Ref. No. 22-0194 KB) and has provided the investigators with written documentation that the study is exempt from further review. Individual informed consent of patients or legal representatives was waived.

## References

[1] Heller AR, Bartenschlager CC, Brunner JO, Marckmann G (2023) „Triagegesetz“ – Regelung mit fatalen Folgen. Anaesthesiologie 72(6):385–394 37233790 10.1007/s00101-023-01286-0 PMC10215064

[2] Zwissler B (2023) „Gut gedacht, schlecht gemacht“ – das „Triage“-Gesetz, seine Defizite und die Folgen. Anaesthesiologie 72(6):381–384 37278745 10.1007/s00101-023-01299-9

[3] Christian MD (2019) Triage. Crit Care Clin 35(4):575–589 31445606 10.1016/j.ccc.2019.06.009 PMC7127292

[4] Michalsen A, Badewien C (2023) New German law: ex-post triage criminalised. ICU Manag Pract 23(1):42–43

[5] Pollaris G, Sabbe M (2016) Reverse triage: more than just another method. Eur J Emerg Med 23(4):240–247 26479736 10.1097/MEJ.0000000000000339

[6] Marckmann G, Neitzke G, Schildmann J, Michalsen A, Dutzmann J, Hartog C et al (2020) Entscheidungen uber die Zuteilung intensivmedizinischer Ressourcen im Kontext der COVID-19-Pandemie: Klinisch-ethische Empfehlungen der DIVI, der DGINA, der DGAI, der DGIIN, der DGNI, der DGP, der DGP und der AEM. Med Klin Intensivmed Notfmed 115(6):477–485 32728769 10.1007/s00063-020-00708-w PMC7387420

[7] Michalsen A, Neitzke G, Dutzmann J, Rogge A, Seidlein AH, Jobges S et al (2021) Overtreatment in intensive care medicine-recognition, designation, and avoidance: position paper of the ethics section of the DIVI and the ethics section of the DGIIN. Med Klin Intensivmed Notfmed 116(4):281–294 33646332 10.1007/s00063-021-00794-4 PMC7919250

[8] Bundesärztekammer (2022) Stellungnahme der Bundesärztekammer zum Referentenentwurf eines Gesetzes zur Änderung des Infektionsschutzgesetzes. https://www.bundesaerztekammer.de/fileadmin/user_upload/BAEK/Politik/Stellungnahmen/20220721_Triage_AEnd_IfSG_SN_BAEK.pdf

[9] COVID-19 AT (2022) Stellungnahme der AWMF Taskforce COVID-19 Leitlinien zum Referentenentwurf eines Gesetzes zur Änderung des Infektionsschutzgesetzes des Bundesministeriums für Gesundheit. https://www.awmf.org/fileadmin/user_upload/dateien/veranstaltungen/stellungnahme-aenderung-infektionsschutzgesetz.pdf

[10] Hick JL, Einav S, Hanfling D, Kissoon N, Dichter JR, Devereaux AV et al (2014) Surge capacity principles: care of the critically ill and injured during pandemics and disasters: CHEST consensus statement. Chest 146(4):e1S–e16S 25144334 10.1378/chest.14-0733

[11] Gaede K, Kubiciel M, Saliger F, Tsambikakis M (2020) Rechtmäßiges Handeln in der dilemmatischen Triage-Entscheidungssituation. MedR 3:129–137

[12] Hörnle T (2023) Ex-Post Triage: Gründe für ihre Zulassung. MedR 9(3):139–142

[13] Bartenschlager CC, Brunner JO, Heller AR (2022) Evaluation von scorebasierten Ansätzen für die Ex-post-Triage auf Intensivstationen während der COVID-19-Pandemie: eine simulationsbasierte Analyse. Notfall Rettungsmed 25(4):221–223 10.1007/s10049-022-01035-7 PMC9073506 35542759

[14] Kubiciel M (2020) Die Triage in der rechtswissenschaftlichen Diskussion. Z Med Ethik 66(4):509–519

[15] Booke H, Booke M (2021) Medical triage during the COVID-19 pandemic: a medical and ethical burden. J Clin Ethics 32(1):73–76 33656459

[16] Cameron J, Savulescu J, Wilkinson D (2021) Is withdrawing treatment really more problematic than withholding treatment? J Med Ethics 47(11):722–726 32451343 10.1136/medethics-2020-106330 PMC7295851

[17] Churpek MM, Gupta S, Spicer AB et al (2021) Machine learning prediction of death in critically ill patients with Coronavirus disease 2019. Crit Care Explor 3(8):e515 34476402 10.1097/CCE.0000000000000515 PMC8378790

[18] Deif MA, Solyman AAA, Alsharif MH, Uthansakul P (2021) Automated triage system for intensive care admissions during the COVID-19 pandemic using hybrid XGboost-AHP approach. Sensors 21(19):6379 34640700 10.3390/s21196379 PMC8512533

[19] Caramello V, Marulli G, Reimondo G, Fanto’ F, Boccuzzi A (2018) Inpatient disposition in overcrowded hospitals: is it safe and effective to use reverse triage and readmission screening tools for appropriate discharge? An observational prospective study of an Italian II level hospital. Int J Clin Pract 73(2):e13281 10.1111/ijcp.13281 30288861

[20] Caramello V, Marulli G, Reimondo G, Fanto’ F, Boccuzzi A (2019) Comparison of reverse triage with national early warning score, sequential organ failure assessment and Charlson comorbidity index to classify medical inpatients of an Italian II level hospital according to their resource’s need. Intern Emerg Med 14(7):1073–1082 30778758 10.1007/s11739-019-02049-9

[21] Kądziołka I, Świstek R, Borowska K, Tyszecki P, Serednicki W (2019) Validation of APACHE II and SAPS II scales at the intensive care unit along with assessment of SOFA scale at the admission as an isolated risk of death predictor. Anaesthesiol Intensive Ther 51(2):107–111 31268271 10.5114/ait.2019.86275

[22] Bai J, Fügener A, Gönsch J, Brunner JO, Blobner M (2021) Managing admission and discharge processes in intensive care units. Health Care Manag Sci 24(4):666–685 34110549 10.1007/s10729-021-09560-6 PMC8189840

[23] Alban A, Chick SE, Dongelmans DA, Vlaar APJ, Sent D (2020) ICU capacity management during the COVID-19 pandemic using a process simulation. Intensive Care Med 46(8):1624–1626 32383060 10.1007/s00134-020-06066-7 PMC7203503

[24] Bai J, Brunner JO, Gerstmeyr S (2020) Simulation and evaluation of ICU management policies, pp 864–875

[25] Wood RM, Pratt AC, Kenward C et al (2021) The value of triage during periods of intense COVID-19 demand: simulation modeling study. Med Decis Making 41(4):393–407 33560181 10.1177/0272989X21994035

[26] Garber S, Brunner JO, Heller AR et al (2023) Simulation of the mortality after different ex ante (secondary) and ex post (tertiary) triage methods in people with disabilities and pre-existing diseases. Anaesthesiologie. 10.1007/s00101-023-01336-7 37733034 10.1007/s00101-023-01336-7 PMC10692011

[27] Le Gall JR, Lemeshow S, Saulnier F (1993) A new simplified acute physiology score (SAPS II) based on a European/north American multicenter study. JAMA 270(24):2957–2963 8254858 10.1001/jama.270.24.2957

[28] Cullen DJ, Civetta JM, Briggs BA, Ferrara LC (1974) Therapeutic intervention scoring system: a method for quantitative comparison of patient care. Crit Care Med 2(2):57–60 4832281

[29] Wehler M, Deetjen P, Gerheuser F, Joachimski F, Schneider H, Wittmann M, Frühwald M (2021) Pandemie Covid-19 – Empfehlungen zur Triage bei Intensivmedizinischer Ressourcenknappheit im Katastrophenfall. In: Hilgendorf E, Hoven E, Rostalski F (eds) Triage in der (Strafrechts‑) Wissenschaft. Nomos, Baden-Baden, pp 361–374

[30] Vincent JL, de Mendonça A, Cantraine F et al (1998) Use of the SOFA score to assess the incidence of organ dysfunction/failure in intensive care units: results of a multicenter, prospective study. Working group on “sepsis-related problems” of the European society of intensive care medicine. Crit Care Med 26(11):1793–1800 9824069 10.1097/00003246-199811000-00016

[31] Wilfong EM, Lovly CM, Gillaspie EA et al (2021) Severity of illness scores at presentation predict ICU admission and mortality in COVID-19. J Emerg Crit Care Med 5:7 34179689 10.21037/jeccm-20-92 PMC8232354

[32] Tukey J (1949) Comparing individual means in the analysis of variance. Biometrics 5(2):99–114 18151955

[33] Nielsen AB, Thorsen-Meyer HC, Belling K et al (2019) Survival prediction in intensive-care units based on aggregation of long-term disease history and acute physiology: a retrospective study of the Danish National Patient Registry and electronic patient records. Lancet Digit Health 1(2):e78–e89 33323232 10.1016/S2589-7500(19)30024-X

[34] Le Gall JR, Neumann A, Hemery F et al (2005) Mortality prediction using SAPS II: an update for French intensive care units. Crit Care 9(6):R645–R652 16280063 10.1186/cc3821 PMC1414016

[35] Péterfi A, Mészáros Á, Szarvas Z et al (2022) Comorbidities and increased mortality of COVID-19 among the elderly: a systematic review. PhysInt. 10.1556/2060.2022.00206 10.1556/2060.2022.00206 35575986

[36] Heller AR, Rössler S, Litz RJ et al (2006) Omega‑3 fatty acids improve the diagnosis-related clinical outcome. Crit Care Med 34(4):972–979 16484909 10.1097/01.CCM.0000206309.83570.45

[37] Mitsch W (2022) Bemerkungen zur „präventiven“ Triage und zur „ex-post“-Triage. Z Int Strafrechtswiss 1(4):323–328

[38] Knochel K, Adaktylos-Surber K, Schmolke EM, Meier LJ, Kuehlmeyer K, Ulm K et al (2022) Preparing for the worst-case scenario in a pandemic: intensivists simulate prioritization and triage of scarce ICU resources. Crit Care Med 50(12):1714–1724 36222541 10.1097/CCM.0000000000005684 PMC9668365

