# Supplementary material for: Evaluation of score-based tertiary triage policies during the COVID-19 pandemic: simulation study with real-world intensive care data
Source: Med Klin Intensivmed Notfmed. 2024 Aug 2;120(4):307–15. doi: 10.1007/s00063-024-01162-8 (PMC12041167; doi:10.1007/s00063-024-01162-8)
Supplement: Supplementary file 1 — The Supplementary Information includes detailed descriptions of the calculation of SAPS II-predictced mortality, of the proceeding of the simulation study and of the results of the simulation study. [file 63_2024_1162_MOESM1_ESM.docx]

# Supplementary Material

Description 1: Calculation of SAPS II-predictced mortality

For the evaluation of our simulation results, based on the SAPS II score, we calculated the SAPS II-predicted mortality rate ${mr}_{b}^{SAPS}$ per patient $b$ as proposed by [27]:

$${mr}_{b}^{SAPS}=\frac{e^{-7.7631+0.0737\cdot{SAPS}_{b}+0.9971\cdot\ln({SAPS}_{b}+1)}}{1+e^{-7.7631+0.0737\cdot{SAPS}_{b}+0.9971\cdot\ln({SAPS}_{b}+1)}}$$

For ${mr}_{b}^{SAPS}<0.5$, the SAPS II-predicted mortality predicts a survival of the patient, i.e., $m_{b}^{SAPS}=0$, and $m_{b}^{SAPS}=1$ otherwise:

$$m_{b}^{SAPS}=\left\{ \begin{aligned} \begin{matrix} 1 & {mr}_{b}^{SAPS} \geq0.5 \end{matrix} \\ \begin{matrix} 0 & {mr}_{b}^{SAPS} <0.5 \end{matrix} \end{aligned} \right.$$

If the SAPS II-predicted mortality rate is smaller than $50\%$, survival of the patient will be predicted. If the SAPS II-predicted mortality rate is bigger than or equal to $50\%$, dead of the patient will be predicted.

Description 2: Proceeding of the simulation study

We predefined the ICU capacity to be the one of the University Hospital of Augsburg, i.e., one occupation of the ICU consisted of $B=60$ patients. In addition, the number of consecutive tertiary triage points in time, $T=3$ and, 6 different scenarios given in Supplementary Table 2 were defined. We considered a maximum number of $T=3$ days due to an ex-ante expected mean ICU-LOS of approx. $4$ days and the assumption of no discharge throughout the time horizon of $T=3$. The 6 scenarios varied in the number of critical care patients $l$ waiting in the queue, $L=\left\{ 12,30,60 \right\}$, and the maximum number of existing intensive care patients subject to tertiary triage, $E=\left\{ 6,12,30 \right\}$.

For a given scenario and simulation run $r\in\left\{ 1,\ldots,R \right\}$, an occupancy of the ICU was generated by randomly sampling $B=60$ patients from the full data set with replacement using the software R. In addition, we randomly generated the currently elapsed time in $t=1$ on the ICU for the $B=60$ patients from a discrete uniform distribution each, i.e., ${ICU-LOS}_{b}^{t=1}\sim\mathrm{UNIF}\left[ 0;\left\lfloor\frac{{ICU-LOS}_{b}}{2} \right\rfloor\right]$. The upper bound $\left\lfloor\frac{{ICU-LOS}_{b}}{2} \right\rfloor$ was set to guarantee an integer solution by the Gaussian brackets and to generate patients on the ICU absolutely in need of intensive care treatment without an option for early discharge. ${ICU-LOS}_{b}$ defined the realized ICU-LOS of patient $b$ and was the basis for the FCFS tertiary triage policy ($p=2$).

For every consecutive point in time $t\in\left\{ 1,\ldots,T \right\}$, $L$ critical care patients waiting in the queue were generated by random sampling with replacement and, for all patients in the queue, the currently elapsed time on the ICU was initialized to be ${ICU-LOS}_{l}^{t}=0$. For every tertiary triage policy $p\in\left\{ 0,\ldots,P \right\}$, the new occupancy of the intensive care unit, the associated mortality on ICU $m_{p,t,r}=\frac{\sum_{b=1}^{B} m_{b,p,t,r}}{B}$ and the associated SAPS II-predicted mortality on ICU $m_{p,t,r}^{SAPS}=\frac{\sum_{b=1}^{B} m_{b,p,t,r}^{SAPS}}{B}$ were calculated automatically. $m_{b,p,t,r}=1$ indicated that patient $b$ per policy $p$, point in time $t$ and simulation run $r$ did not survive. At the end of every point in time $t$, the currently elapsed time for all patients on the ICU was updated, i.e. ${ICU-LOS}_{b}^{t+1}={ICU-LOS}_{b}^{t}+1$.

Finally for the scenario, the average mortality $m_{p,t}=\frac{m_{p,t,r}}{R}$, the average SAPS II-predicted mortality $m_{p,t}^{SAPS}=\frac{m_{p,t,r}^{SAPS}}{R}$ and the $T=3$ Analyses of Variance (ANOVAs) including boxplots as well as post hoc tests on a 5 % significance level were provided (see Supplementary Figure 2). We applied Tukey's HSD (honestly significant difference) post hoc test, a multivariate multiple comparison procedure [32]. A policy was assumed to be superior if and only if $m_{p,t}<m_{q,t}$ with $p,q\in\left\{ 0,\ldots,9 \right\}$ and $p\neq q$. For an optimal policy, it holds that the average mortality on ICU is minimized, i.e., $p_{t}^{*}=\mathrm{argmin}_{p} m_{p,t} \forall t\in\left\{ 1,\ldots,T \right\}$. Based on the hypothesis tests, we evaluated the actual inferential significance of differences in between the policies. Please find an overview of important indices and parameters in Supplementary Table 3.

Table 1: Calculation of original DIVI score by the sum per criterion (upper table, SOFA=Sequential Organ Failure Assessment) and of adjusted DIVI score for this study by the sum per criterion (lower table, SAPS II= Simplified Acute Physiology Score II)

| Criterion | Points | | | | |
| --- | --- | --- | --- | --- | --- |
|  | 1 | 2 | 3 | 4 |  |
| SOFA | < 6 | 6-8 | 9-11 | > 12 |  |
| Prognosis limiting factors |  | severe |  | critical |  |
| (Secondary) criterion age | < 50 | 50-69 | 70-84 | ≥ 85 |  |

| Criterion | Points | | | | |
| --- | --- | --- | --- | --- | --- |
|  | 1 | 2 | 3 | 4 |  |
| SAPS II | < 41 | 41-54 | 55-75 | > 75 |  |
| No. secondary diagnoses |  | 13-24 |  | > 24 |  |
| (Optional) criterion age | < 50 | 50-69 | 70-84 | ≥ 85 |  |

Table 2: Definition of the different scenarios evaluated by the simulation study

| Scenario | 1 | 2 | 3 | 4 | 5 | 6 |
| --- | --- | --- | --- | --- | --- | --- |
| No. of simulation runs: $R$ | 1,000 | 1,000 | 1,000 | 1,000 | 1,000 | 1,000 |
| No. of policies: $P+1$ | 10 | 10 | 10 | 10 | 10 | 10 |
| ICU capacity [patients]: $B$ | 60 | 60 | 60 | 60 | 60 | 60 |
| No. of consecutive ex-post triage points in time [days]: $T$ | 3 | 3 | 3 | 3 | 3 | 3 |
| Length of the queue [patients]: $L$ | 12 | 30 | 30 | 60 | 60 | 60 |
| Max. no. of patients subject to ex-post triage: $E$ | 6 | 6 | 12 | 6 | 12 | 30 |

Table 3: Important indices and parameters

| Description | Index $\in$ set and/or parameters |
| --- | --- |
| Patient on ICU | $b\in\left\{ 1,\ldots,B \right\}$ |
| Policy | $p\in\left\{ 1,\ldots,P \right\}$ |
| Simulation run | $r\in\left\{ 1,\ldots,R \right\}$ |
| Points in time | $t\in\left\{ 1,\ldots,T \right\}$ |
| ICU capacity [patients] | $B$ |
| Mortality on ICU per $b,p$, $t$, $r$ | $m_{b,p,t,r}$ |
| Mortality on ICU per $p$, $t$, $r$ | $m_{p,t,r}$ |
| (Average) mortality on ICU per $p$, $t$ | $m_{p,t}$ |

Table 4: Descriptive statistics of the data set (SD=Standard deviation, Max=Maximum, Min=Minimum, $m_{b}^{SAPS}$: SAPS II-predicted mortality)

|  |  | All patients | COVID-19 patients | Non-COVID-19 patients | Surviving patients | Non-surviving patients |
| --- | --- | --- | --- | --- | --- | --- |
| Number of patients | No. | 1,083 | 247 | 836 | 825 | 258 |
| Age [years] | Mean | 64.58 | 61.09 | 65.61 | 63.08 | 69.38 |
|  | SD | 15.72 | 15.47 | 15.66 | 16.25 | 12.80 |
|  | Max | 96.00 | 92.00 | 96.00 | 96.00 | 94.00 |
|  | Min | 16.00 | 17.00 | 16.00 | 16.00 | 26.00 |
| TISS | Mean | 9.44 | 9.46 | 9.44 | 8.66 | 11.94 |
|  | SD | 5.41 | 4.19 | 5.72 | 5.26 | 5.14 |
|  | Max | 35.00 | 24.00 | 35.00 | 35.00 | 34.00 |
|  | Min | 0.00 | 0.00 | 0.00 | 0.00 | 0.00 |
| SAPS II | Mean | 35.01 | 33.94 | 35.32 | 32.18 | 44.04 |
|  | SD | 12.70 | 11.63 | 12.98 | 11.33 | 12.63 |
|  | Max | 79.00 | 71.00 | 79.00 | 71.00 | 79.00 |
|  | Min | 6.00 | 8.00 | 6.00 | 6.00 | 11.00 |
| Ratio of patients with $m_{b}^{SAPS}=1$ [%] | Ratio | 10.25 | 7.29 | 11.12 | 5.21 | 26.36 |
| ICU-LOS [days] | Mean | 3.99 | 5.68 | 3.49 | 3.75 | 4.74 |
|  | SD | 5.39 | 6.87 | 4.76 | 5.26 | 5.75 |
|  | Max | 48.00 | 42.00 | 48.00 | 48.00 | 38.00 |
|  | Min | 1.00 | 1.00 | 1.00 | 1.00 | 1.00 |
| Hospital LOS [days] | Mean | 17.77 | 17.54 | 17.83 | 18.60 | 15.08 |
|  | SD | 13.57 | 13.35 | 13.64 | 13.40 | 13.76 |
|  | Max | 105.00 | 90.00 | 105.00 | 105.00 | 101.00 |
|  | Min | 2.00 | 2.00 | 2.00 | 2.00 | 2.00 |
| Secondary diagnosis [No.] | Mean | 12.21 | 10.18 | 12.81 | 11.51 | 14.46 |
|  | SD | 6.43 | 5.43 | 6.58 | 6.25 | 6.48 |
|  | Max | 50.00 | 34.00 | 50.00 | 40.00 | 50.00 |
|  | Min | 0.00 | 2.00 | 0.00 | 1.00 | 0.00 |

Table 5: Simulation-based average values of the mortality on ICU for the different scenarios, points in time $t$ and policies $p$. Grey color marks the minimum per respective row (scenario).

| $t=1$ | $p$ | 0 | 1 | 2 | 3 | 4 | 5 | 6 | 7 | 8 | 9 |
| --- | --- | --- | --- | --- | --- | --- | --- | --- | --- | --- | --- |
|  | Scenario 1 | 0.24 | 0.24 | 0.24 | 0.23 | 0.19 | 0.21 | 0.22 | 0.23 | 0.21 | 0.21 |
|  | Scenario 2 | 0.24 | 0.24 | 0.24 | 0.22 | 0.18 | 0.21 | 0.21 | 0.23 | 0.20 | 0.20 |
|  | Scenario 3 | 0.24 | 0.24 | 0.24 | 0.20 | 0.15 | 0.18 | 0.20 | 0.22 | 0.18 | 0.18 |
|  | Scenario 4 | 0.24 | 0.24 | 0.24 | 0.21 | 0.18 | 0.20 | 0.21 | 0.22 | 0.20 | 0.20 |
|  | Scenario 5 | 0.24 | 0.24 | 0.24 | 0.20 | 0.15 | 0.18 | 0.18 | 0.22 | 0.18 | 0.17 |
|  | Scenario 6 | 0.24 | 0.24 | 0.24 | 0.17 | 0.11 | 0.15 | 0.16 | 0.21 | 0.12 | 0.12 |
| $t=2$ | $p$ | 0 | 1 | 2 | 3 | 4 | 5 | 6 | 7 | 8 | 9 |
|  | Scenario 1 | 0.24 | 0.24 | 0.23 | 0.21 | 0.16 | 0.19 | 0.20 | 0.22 | 0.19 | 0.19 |
|  | Scenario 2 | 0.24 | 0.24 | 0.23 | 0.20 | 0.15 | 0.18 | 0.18 | 0.22 | 0.18 | 0.17 |
|  | Scenario 3 | 0.24 | 0.24 | 0.23 | 0.18 | 0.11 | 0.15 | 0.17 | 0.21 | 0.14 | 0.13 |
|  | Scenario 4 | 0.24 | 0.24 | 0.23 | 0.19 | 0.14 | 0.17 | 0.17 | 0.22 | 0.18 | 0.17 |
|  | Scenario 5 | 0.24 | 0.24 | 0.23 | 0.17 | 0.09 | 0.14 | 0.14 | 0.21 | 0.13 | 0.12 |
|  | Scenario 6 | 0.24 | 0.24 | 0.23 | 0.16 | 0.07 | 0.11 | 0.13 | 0.21 | 0.10 | 0.09 |
| $t=3$ | $p$ | 0 | 1 | 2 | 3 | 4 | 5 | 6 | 7 | 8 | 9 |
|  | Scenario 1 | 0.24 | 0.24 | 0.23 | 0.20 | 0.14 | 0.17 | 0.19 | 0.21 | 0.17 | 0.16 |
|  | Scenario 2 | 0.24 | 0.24 | 0.23 | 0.18 | 0.12 | 0.15 | 0.16 | 0.21 | 0.16 | 0.15 |
|  | Scenario 3 | 0.24 | 0.24 | 0.23 | 0.16 | 0.09 | 0.13 | 0.14 | 0.21 | 0.10 | 0.10 |
|  | Scenario 4 | 0.24 | 0.24 | 0.23 | 0.17 | 0.11 | 0.15 | 0.15 | 0.21 | 0.15 | 0.14 |
|  | Scenario 5 | 0.24 | 0.24 | 0.23 | 0.15 | 0.06 | 0.11 | 0.10 | 0.21 | 0.10 | 0.09 |
|  | Scenario 6 | 0.24 | 0.24 | 0.24 | 0.15 | 0.06 | 0.10 | 0.09 | 0.21 | 0.10 | 0.09 |

Table 6: Simulation-based average values of the SAPS II-predicted mortality on ICU for the different scenarios, points in time t and policies p. Grey color marks the minimum per respective row (scenario).

| $t=1$ | $p$ | 0 | 1 | 2 | 3 | 4 | 5 | 6 | 7 | 8 | 9 |
| --- | --- | --- | --- | --- | --- | --- | --- | --- | --- | --- | --- |
|  | Scenario 1 | 0.10 | 0.10 | 0.10 | 0.06 | 0.00 | 0.05 | 0.06 | 0.13 | 0.01 | 0.01 |
|  | Scenario 2 | 0.10 | 0.10 | 0.10 | 0.09 | 0.02 | 0.07 | 0.08 | 0.11 | 0.05 | 0.06 |
|  | Scenario 3 | 0.10 | 0.10 | 0.10 | 0.08 | 0.00 | 0.06 | 0.07 | 0.12 | 0.04 | 0.04 |
|  | Scenario 4 | 0.10 | 0.10 | 0.10 | 0.09 | 0.02 | 0.07 | 0.08 | 0.11 | 0.05 | 0.06 |
|  | Scenario 5 | 0.10 | 0.10 | 0.10 | 0.07 | 0.00 | 0.06 | 0.06 | 0.12 | 0.04 | 0.04 |
|  | Scenario 6 | 0.10 | 0.10 | 0.10 | 0.06 | 0.00 | 0.05 | 0.06 | 0.13 | 0.01 | 0.01 |
| $t=2$ | $p$ | 0 | 1 | 2 | 3 | 4 | 5 | 6 | 7 | 8 | 9 |
|  | Scenario 1 | 0.10 | 0.10 | 0.10 | 0.08 | 0.00 | 0.06 | 0.07 | 0.11 | 0.04 | 0.04 |
|  | Scenario 2 | 0.10 | 0.10 | 0.11 | 0.07 | 0.00 | 0.06 | 0.06 | 0.11 | 0.04 | 0.04 |
|  | Scenario 3 | 0.10 | 0.10 | 0.11 | 0.06 | 0.00 | 0.04 | 0.06 | 0.12 | 0.02 | 0.01 |
|  | Scenario 4 | 0.10 | 0.10 | 0.11 | 0.07 | 0.00 | 0.06 | 0.06 | 0.11 | 0.04 | 0.04 |
|  | Scenario 5 | 0.10 | 0.10 | 0.11 | 0.05 | 0.00 | 0.04 | 0.04 | 0.12 | 0.02 | 0.01 |
|  | Scenario 6 | 0.10 | 0.10 | 0.11 | 0.04 | 0.00 | 0.03 | 0.04 | 0.13 | 0.00 | 0.00 |
| $t=3$ | $p$ | 0 | 1 | 2 | 3 | 4 | 5 | 6 | 7 | 8 | 9 |
|  | Scenario 1 | 0.10 | 0.10 | 0.11 | 0.07 | 0.00 | 0.05 | 0.07 | 0.12 | 0.03 | 0.03 |
|  | Scenario 2 | 0.10 | 0.10 | 0.11 | 0.06 | 0.00 | 0.05 | 0.05 | 0.12 | 0.03 | 0.02 |
|  | Scenario 3 | 0.10 | 0.10 | 0.11 | 0.05 | 0.00 | 0.04 | 0.05 | 0.13 | 0.00 | 0.00 |
|  | Scenario 4 | 0.10 | 0.10 | 0.11 | 0.06 | 0.00 | 0.05 | 0.05 | 0.12 | 0.03 | 0.02 |
|  | Scenario 5 | 0.10 | 0.10 | 0.11 | 0.04 | 0.00 | 0.03 | 0.03 | 0.13 | 0.00 | 0.00 |
|  | Scenario 6 | 0.10 | 0.10 | 0.10 | 0.04 | 0.00 | 0.03 | 0.02 | 0.13 | 0.00 | 0.00 |

Table 7: (Adjusted) $p$-values of the F tests (F) and pairwise post hoc tests for the different scenarios and points in time $t$. For example, 1-0 indicates the pairwise inferential statistical comparison of policy $p=1$ and $p=0$. The significance level is set to 5 %. Grey color marks a significant result.

|  | Scenario 1 | | | Scenario 2 | | | Scenario 3 | | | Scenario 4 | | | Scenario 5 | | | Scenario 6 | | |
| --- | --- | --- | --- | --- | --- | --- | --- | --- | --- | --- | --- | --- | --- | --- | --- | --- | --- | --- |
| $t$ | 1 | 2 | 3 | 1 | 2 | 3 | 1 | 2 | 3 | 1 | 2 | 3 | 1 | 2 | 3 | 1 | 2 | 3 |
| F | 0.00 | 0.00 | 0.00 | 0.00 | 0.00 | 0.00 | 0.00 | 0.00 | 0.00 | 0.00 | 0.00 | 0.00 | 0.00 | 0.00 | 0.00 | 0.00 | 0.00 | 0.00 |
| 1-0 | 1.00 | 1.00 | 1.00 | 1.00 | 1.00 | 1.00 | 1.00 | 1.00 | 0.99 | 1.00 | 1.00 | 1.00 | 1.00 | 1.00 | 1.00 | 1.00 | 1.00 | 1.00 |
| 2-0 | 1.00 | 0.00 | 0.00 | 1.00 | 0.12 | 0.00 | 1.00 | 0.00 | 0.02 | 1.00 | 0.01 | 0.00 | 1.00 | 0.00 | 0.00 | 0.95 | 0.03 | 1.00 |
| 3-0 | 0.00 | 0.00 | 0.00 | 0.00 | 0.00 | 0.00 | 0.00 | 0.00 | 0.00 | 0.00 | 0.00 | 0.00 | 0.00 | 0.00 | 0.00 | 0.00 | 0.00 | 0.00 |
| 4-0 | 0.00 | 0.00 | 0.00 | 0.00 | 0.00 | 0.00 | 0.00 | 0.00 | 0.00 | 0.00 | 0.00 | 0.00 | 0.00 | 0.00 | 0.00 | 0.00 | 0.00 | 0.00 |
| 5-0 | 0.00 | 0.00 | 0.00 | 0.00 | 0.00 | 0.00 | 0.00 | 0.00 | 0.00 | 0.00 | 0.00 | 0.00 | 0.00 | 0.00 | 0.00 | 0.00 | 0.00 | 0.00 |
| 6-0 | 0.00 | 0.00 | 0.00 | 0.00 | 0.00 | 0.00 | 0.00 | 0.00 | 0.00 | 0.00 | 0.00 | 0.00 | 0.00 | 0.00 | 0.00 | 0.00 | 0.00 | 0.00 |
| 7-0 | 0.00 | 0.00 | 0.00 | 0.00 | 0.00 | 0.00 | 0.00 | 0.00 | 0.00 | 0.00 | 0.00 | 0.00 | 0.00 | 0.00 | 0.00 | 0.00 | 0.00 | 0.00 |
| 8-0 | 0.00 | 0.00 | 0.00 | 0.00 | 0.00 | 0.00 | 0.00 | 0.00 | 0.00 | 0.00 | 0.00 | 0.00 | 0.00 | 0.00 | 0.00 | 0.00 | 0.00 | 0.00 |
| 9-0 | 0.00 | 0.00 | 0.00 | 0.00 | 0.00 | 0.00 | 0.00 | 0.00 | 0.00 | 0.00 | 0.00 | 0.00 | 0.00 | 0.00 | 0.00 | 0.00 | 0.00 | 0.00 |
| 2-1 | 1.00 | 0.00 | 0.00 | 1.00 | 0.16 | 0.00 | 1.00 | 0.00 | 0.29 | 1.00 | 0.02 | 0.00 | 1.00 | 0.00 | 0.01 | 1.00 | 0.05 | 1.00 |
| 3-1 | 0.00 | 0.00 | 0.00 | 0.00 | 0.00 | 0.00 | 0.00 | 0.00 | 0.00 | 0.00 | 0.00 | 0.00 | 0.00 | 0.00 | 0.00 | 0.00 | 0.00 | 0.00 |
| 4-1 | 0.00 | 0.00 | 0.00 | 0.00 | 0.00 | 0.00 | 0.00 | 0.00 | 0.00 | 0.00 | 0.00 | 0.00 | 0.00 | 0.00 | 0.00 | 0.00 | 0.00 | 0.00 |
| 5-1 | 0.00 | 0.00 | 0.00 | 0.00 | 0.00 | 0.00 | 0.00 | 0.00 | 0.00 | 0.00 | 0.00 | 0.00 | 0.00 | 0.00 | 0.00 | 0.00 | 0.00 | 0.00 |
| 6-1 | 0.00 | 0.00 | 0.00 | 0.00 | 0.00 | 0.00 | 0.00 | 0.00 | 0.00 | 0.00 | 0.00 | 0.00 | 0.00 | 0.00 | 0.00 | 0.00 | 0.00 | 0.00 |
| 7-1 | 0.00 | 0.00 | 0.00 | 0.00 | 0.00 | 0.00 | 0.00 | 0.00 | 0.00 | 0.00 | 0.00 | 0.00 | 0.00 | 0.00 | 0.00 | 0.00 | 0.00 | 0.00 |
| 8-1 | 0.00 | 0.00 | 0.00 | 0.00 | 0.00 | 0.00 | 0.00 | 0.00 | 0.00 | 0.00 | 0.00 | 0.00 | 0.00 | 0.00 | 0.00 | 0.00 | 0.00 | 0.00 |
| 9-1 | 0.00 | 0.00 | 0.00 | 0.00 | 0.00 | 0.00 | 0.00 | 0.00 | 0.00 | 0.00 | 0.00 | 0.00 | 0.00 | 0.00 | 0.00 | 0.00 | 0.00 | 0.00 |
| 3-2 | 0.00 | 0.00 | 0.00 | 0.00 | 0.00 | 0.00 | 0.00 | 0.00 | 0.00 | 0.00 | 0.00 | 0.00 | 0.00 | 0.00 | 0.00 | 0.00 | 0.00 | 0.00 |
| 4-2 | 0.00 | 0.00 | 0.00 | 0.00 | 0.00 | 0.00 | 0.00 | 0.00 | 0.00 | 0.00 | 0.00 | 0.00 | 0.00 | 0.00 | 0.00 | 0.00 | 0.00 | 0.00 |
| 5-2 | 0.00 | 0.00 | 0.00 | 0.00 | 0.00 | 0.00 | 0.00 | 0.00 | 0.00 | 0.00 | 0.00 | 0.00 | 0.00 | 0.00 | 0.00 | 0.00 | 0.00 | 0.00 |
| 6-2 | 0.00 | 0.00 | 0.00 | 0.00 | 0.00 | 0.00 | 0.00 | 0.00 | 0.00 | 0.00 | 0.00 | 0.00 | 0.00 | 0.00 | 0.00 | 0.00 | 0.00 | 0.00 |
| 7-2 | 0.00 | 0.00 | 0.00 | 0.00 | 0.00 | 0.00 | 0.00 | 0.00 | 0.00 | 0.00 | 0.00 | 0.00 | 0.00 | 0.00 | 0.00 | 0.00 | 0.00 | 0.00 |
| 8-2 | 0.00 | 0.00 | 0.00 | 0.00 | 0.00 | 0.00 | 0.00 | 0.00 | 0.00 | 0.00 | 0.00 | 0.00 | 0.00 | 0.00 | 0.00 | 0.00 | 0.00 | 0.00 |
| 9-2 | 0.00 | 0.00 | 0.00 | 0.00 | 0.00 | 0.00 | 0.00 | 0.00 | 0.00 | 0.00 | 0.00 | 0.00 | 0.00 | 0.00 | 0.00 | 0.00 | 0.00 | 0.00 |
| 4-3 | 0.00 | 0.00 | 0.00 | 0.00 | 0.00 | 0.00 | 0.00 | 0.00 | 0.00 | 0.00 | 0.00 | 0.00 | 0.00 | 0.00 | 0.00 | 0.00 | 0.00 | 0.00 |
| 5-3 | 0.00 | 0.00 | 0.00 | 0.00 | 0.00 | 0.00 | 0.00 | 0.00 | 0.00 | 0.00 | 0.00 | 0.00 | 0.00 | 0.00 | 0.00 | 0.00 | 0.00 | 0.00 |
| 6-3 | 1.00 | 0.00 | 0.00 | 0.68 | 0.00 | 0.00 | 0.01 | 0.00 | 0.00 | 0.38 | 0.00 | 0.00 | 0.00 | 0.00 | 0.00 | 0.00 | 0.00 | 0.00 |
| 7-3 | 1.00 | 0.14 | 0.00 | 0.01 | 0.00 | 0.00 | 0.00 | 0.00 | 0.00 | 0.00 | 0.00 | 0.00 | 0.00 | 0.00 | 0.00 | 0.00 | 0.00 | 0.00 |
| 8-3 | 0.00 | 0.00 | 0.00 | 0.00 | 0.00 | 0.00 | 0.00 | 0.00 | 0.00 | 0.00 | 0.00 | 0.00 | 0.00 | 0.00 | 0.00 | 0.00 | 0.00 | 0.00 |
| 9-3 | 0.00 | 0.00 | 0.00 | 0.00 | 0.00 | 0.00 | 0.00 | 0.00 | 0.00 | 0.00 | 0.00 | 0.00 | 0.00 | 0.00 | 0.00 | 0.00 | 0.00 | 0.00 |
| 5-4 | 0.00 | 0.00 | 0.00 | 0.00 | 0.00 | 0.00 | 0.00 | 0.00 | 0.00 | 0.00 | 0.00 | 0.00 | 0.00 | 0.00 | 0.00 | 0.00 | 0.00 | 0.00 |
| 6-4 | 0.00 | 0.00 | 0.00 | 0.00 | 0.00 | 0.00 | 0.00 | 0.00 | 0.00 | 0.00 | 0.00 | 0.00 | 0.00 | 0.00 | 0.00 | 0.00 | 0.00 | 0.00 |
| 7-4 | 0.00 | 0.00 | 0.00 | 0.00 | 0.00 | 0.00 | 0.00 | 0.00 | 0.00 | 0.00 | 0.00 | 0.00 | 0.00 | 0.00 | 0.00 | 0.00 | 0.00 | 0.00 |
| 8-4 | 0.00 | 0.00 | 0.00 | 0.00 | 0.00 | 0.00 | 0.00 | 0.00 | 0.00 | 0.00 | 0.00 | 0.00 | 0.00 | 0.00 | 0.00 | 0.00 | 0.00 | 0.00 |
| 9-4 | 0.00 | 0.00 | 0.00 | 0.00 | 0.00 | 0.00 | 0.00 | 0.00 | 0.00 | 0.00 | 0.00 | 0.00 | 0.00 | 0.00 | 0.00 | 0.00 | 0.00 | 0.00 |
| 6-5 | 0.00 | 0.00 | 0.00 | 0.20 | 0.18 | 0.00 | 0.00 | 0.00 | 0.00 | 0.75 | 0.99 | 1.00 | 0.80 | 1.00 | 0.26 | 0.00 | 0.00 | 0.00 |
| 7-5 | 0.00 | 0.00 | 0.00 | 0.00 | 0.00 | 0.00 | 0.00 | 0.00 | 0.00 | 0.00 | 0.00 | 0.00 | 0.00 | 0.00 | 0.00 | 0.00 | 0.00 | 0.00 |
| 8-5 | 0.98 | 0.68 | 0.82 | 1.00 | 1.00 | 1.00 | 0.36 | 0.00 | 0.00 | 1.00 | 0.84 | 0.01 | 1.00 | 0.44 | 0.00 | 0.00 | 0.00 | 0.97 |
| 9-5 | 0.87 | 0.15 | 0.00 | 0.71 | 0.03 | 0.01 | 0.02 | 0.00 | 0.00 | 0.98 | 0.77 | 0.49 | 0.07 | 0.00 | 0.00 | 0.00 | 0.00 | 0.00 |
| 7-6 | 0.93 | 0.00 | 0.00 | 0.00 | 0.00 | 0.00 | 0.00 | 0.00 | 0.00 | 0.00 | 0.00 | 0.00 | 0.00 | 0.00 | 0.00 | 0.00 | 0.00 | 0.00 |
| 8-6 | 0.00 | 0.00 | 0.00 | 0.02 | 0.02 | 0.01 | 0.00 | 0.00 | 0.00 | 0.80 | 1.00 | 0.18 | 0.67 | 0.27 | 0.36 | 0.00 | 0.00 | 0.07 |
| 9-6 | 0.00 | 0.00 | 0.00 | 0.00 | 0.00 | 0.00 | 0.00 | 0.00 | 0.00 | 0.11 | 0.14 | 0.07 | 0.00 | 0.00 | 0.00 | 0.00 | 0.00 | 0.00 |
| 8-7 | 0.00 | 0.00 | 0.00 | 0.00 | 0.00 | 0.00 | 0.00 | 0.00 | 0.00 | 0.00 | 0.00 | 0.00 | 0.00 | 0.00 | 0.00 | 0.00 | 0.00 | 0.00 |
| 9-7 | 0.00 | 0.00 | 0.00 | 0.00 | 0.00 | 0.00 | 0.00 | 0.00 | 0.00 | 0.00 | 0.00 | 0.00 | 0.00 | 0.00 | 0.00 | 0.00 | 0.00 | 0.00 |
| 9-8 | 1.00 | 1.00 | 0.43 | 0.99 | 0.24 | 0.00 | 0.98 | 0.12 | 1.00 | 0.97 | 0.03 | 0.00 | 0.12 | 0.00 | 0.00 | 0.81 | 0.17 | 0.00 |


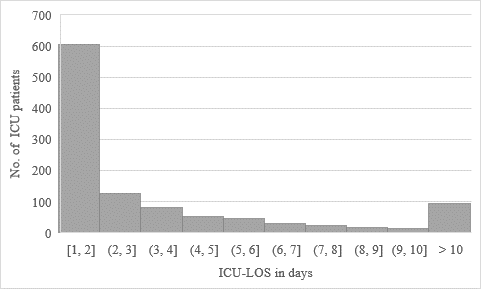


Figure 1: Histogram of ICU-LOS


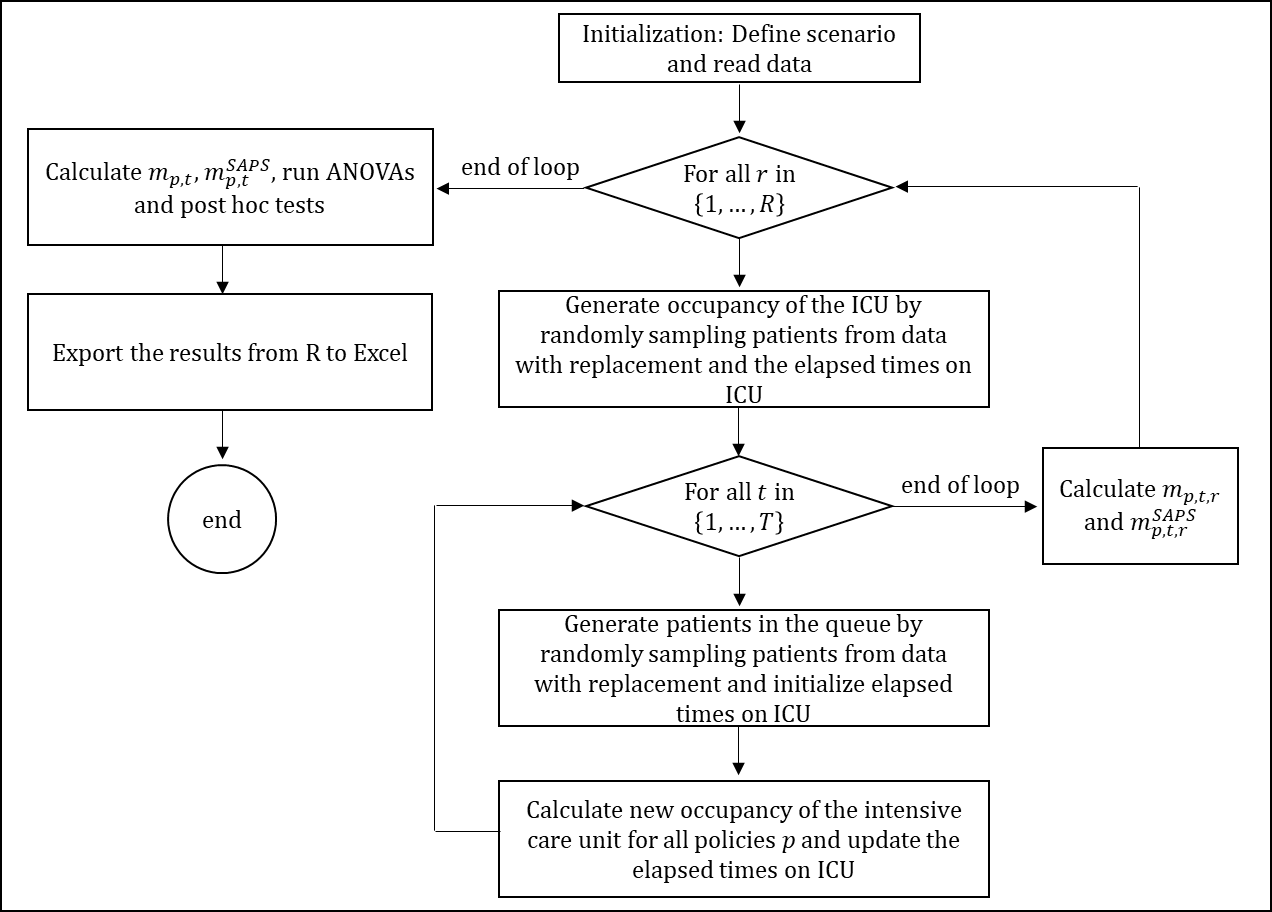


Figure 2: Flowchart of R-based simulation for a given scenario

References for Supplementary (numbered as in the main document):

[27] Le Gall JR, Lemeshow S, Saulnier F. A new Simplified Acute Physiology Score (SAPS II) based on a European/North American multicenter study [published correction appears in JAMA 1994 May 4;271(17):1321]. *JAMA*. 1993;270(24):2957-2963.

[32] Tukey J. Comparing Individual Means in the Analysis of Variance. *Biometrics*. 1949;5(2):99-114.
